# Explainable Artificial Intelligence-Based IoT Device Malware Detection Mechanism Using Image Visualization and Fine-Tuned CNN-Based Transfer Learning Model

**DOI:** 10.1155/2022/7671967

**Published:** 2022-07-15

**Authors:** Hamad Naeem, Bandar M. Alshammari, Farhan Ullah

**Affiliations:** ^1^School of Computer Science and Technology, Zhoukou Normal University, Zhoukou 466001, Henan, China; ^2^School of Computer and Information Sciences, Jouf University, Sakakah, Saudi Arabia; ^3^School of Software, Northwestern Polytechnical University, Xian, 710072, Shaanxi, China

## Abstract

Automated malware detection is a prominent issue in the world of network security because of the rising number and complexity of malware threats. It is time-consuming and resource intensive to manually analyze all malware files in an application using traditional malware detection methods. Polymorphism and code obfuscation were created by malware authors to bypass the standard signature-based detection methods used by antivirus vendors. Malware detection using deep learning (DL) approaches has recently been implemented in an effort to address this problem. This study compares the detection of IoT device malware using three current state-of-the-art CNN models that have been pretrained. Large-scale learning performance using GNB, SVM, DT, LR, K-NN, and ensemble classifiers with CNN models is also included in the results. In light of the findings, a pretrained Inception-v3 CNN-based transfer learned model with fine-tuned strategy is proposed to identify IoT device malware by utilizing color image malware display of android Dalvik Executable File (DEX). Inception-v3 retrieves the malware's most important features. After that, a global max-pooling layer is applied, and a SoftMax classifier is used to classify the features. Finally, gradient-weighted class activation mapping (Grad-CAM) along the t-distributed stochastic neighbor embedding (t-SNE) is used to understand the overall performance of the proposed method. The proposed method achieved an accuracy of 98.5% and 91%, respectively, in the binary and multiclass prediction of malware images from IoT devices, exceeding the comparison methods in different evaluation parameters.

## 1. Introduction

**Figure 1:**
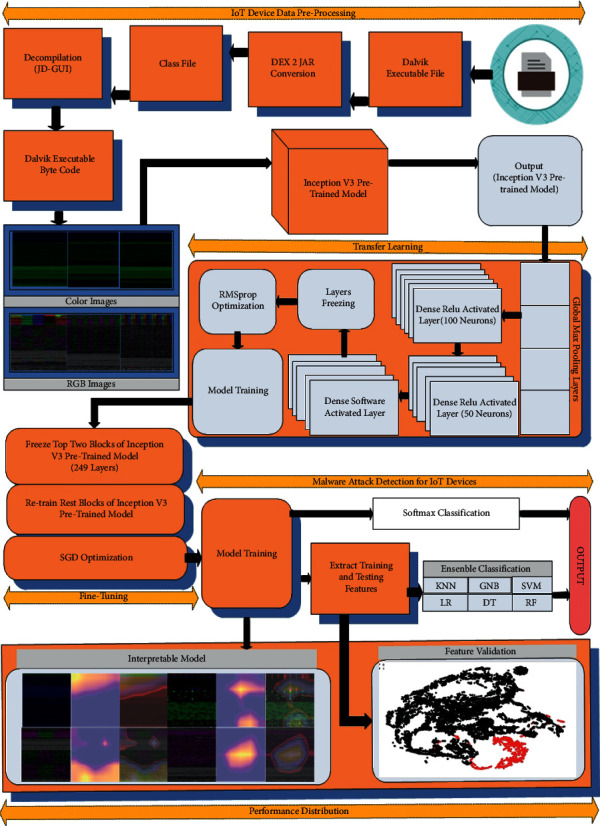
Explainable IoT device malware classification framework.

Malware is an intrusive program that is designed to attack computers and steal personal information without the user's permission. A computer system's privacy can be infiltrated by malware such as adware, spyware, rootkits, and Trojan horses. In 2020, Kaspersky Lab discovered 5,683,694 suspicious android apks, which is 2,179,742 additional suspicious apks than were discovered in 2019. The data for the years 2017 to 2020 are shown in Figure 1. As of May 2021, android had monthly more than three billion active individuals, producing it the biggest and most popular operating system. According to the Forrester study, Android accounts for 74% of the smartphone market, while iOS accounts for 21%. According to IDC's Global Quarterly Smartphone Tracking Service, approximately 7.7% more smartphones shipped in 2021 than in 2020. Another important reason why Android is more vulnerable to malware than iOS is the dispersion of Android devices over previous editions. In 2019, Google has announced that it would no longer provide security patches for Android OS version 6.0 and former versions. According to data statistics of Google, most Android devices fall within this group. Twenty percent of Android phones are running the most recent eleventh version of Android OS, and Android's older versions are the main targets for cyberattacks. Digital banking is now more popular than ever due to the COVID-19 pandemic, and a sizable portion of those clients prefers mobile banking services. The number of online banking Trojans is increased from 69,777 in 2019 to 156,710 in 2020. These data clearly show that attackers are interested in banking data, and malware detection is critical. Malware detection and prevention are critical for ensuring that clients are not at risk of data breaches. The most common malware analysis strategies are static, dynamic, and hybrid [1]. When performing a static analysis, users examine the files rather than running the code. Obfuscation methods and dynamic code packing may have an impact on the operation of static analysis. This is because it monitors the program while it is operating in a sandbox and gathers behavioral data from the running applications. Dynamic analysis can better handle code obfuscation. Nonetheless, the time of examination and resource overhead are major drawbacks of this method. Furthermore, executing an application in a sandbox to cover all possible options may be impossible. Besides that, some malware attacks that can recognize sandbox may not exhibit malicious behavior during dynamic execution. Cross analysis utilizes combine functionalities of both abovementioned analysis. Its main disadvantage is that it consumes a lot of resources and takes a long time to analyze. The Android malware analysis data are used to identify Android malware, which has been gathered [2,3].

Because of the rapid growth of malware variants and advances in machine learning, machine learning-based malware analysis has grown in popularity (ML). Machine learning-based approaches have a higher detection rate and can detect previously unknown malware [4]. However, these methods are heavily reliant on feature engineering, which necessitates a high level of expertise and is time extensive. Deep learning (DL) approaches have recently supplanted conventional techniques for detecting Android malware [5]. By learning feature representation, these approaches alleviate the need for human involvement in feature extraction, screening, and representation. Image-based methods are obtaining popularity these days [6].

The most critical concerns in the fields of machine intelligence and malware analysis are as follows: classification with the highest predictive performance is difficult to achieve since malicious code variants are more similar. Detecting obfuscated or encrypted malware samples using typical malware detection methods such as static, dynamic, hybrid, and image analyses is extremely difficult. In order to achieve the solution of above problems, the proposed work introduces a visualization-based detection strategy. The ability to distinguish between various sections of the malware binary by visualizing it as a colorful image is an advantage of malware writers altering only a small section of the virus codes in order to develop a new mutant. RGB malware images are created from malicious executables and benign apps. Images-based malware models do not need feature engineering, build rapidly, and are resistant to code obfuscation; therefore, they are ideal for malware detection. Then, there is the fact that they are platform independent, which means they can be used on any operating system, not just Android.

This study has the following contributions:

- A transfer learning-based pretrained CNN model with fine-tuning is proposed for IoT device malware classification.
- To improve neural network performance and decrease the need for a large amount of data training, transfer learning is developed by adding RMSprop optimization (root mean square), global max pooling, and dense activated layers with a pretrained CNN model.
- To overcome the challenge of limited data availability for a new deep learning model, fine-tuning is proposed by freezing a few blocks and retraining the rest blocks of the pretrained CNN model with stochastic gradient descent (SGD) optimization.
- Gradient weighted class activation mapping (Grad-CAM) is used to create cumulative heatmaps that help the security analyst better understand the classifier choice. Analysts can use the cumulative heatmap to assess the model's reliability in a visual manner. Furthermore, t-distributed stochastic neighbor embedding (t-SNE) is used to validate the density of features retrieved from the proposed transfer learning-based pretrained CNN model.
- To test the effectiveness of IoT device malware classification, we learn large-scale data with GNB, SVM, DT, LR, and K-NN classifiers, as well as ensemble classifiers in combination with CNN models. Besides this, the experimental outcomes of the proposed transfer learning-based CNN pretrained model are compared with other state-of-the-art pretrained CNN models.

This study is arranged as follows: Section 2 provides a review of the literature, Section 3 explains the suggested strategy, Section 4 presents results and discussions, Section 5 displays the performance validation of proposed method, and Section 6 concludes the study.

## 2. Literature Review

Because of evolutions in CNNs, computer vision approaches have recently received a lot of attention for a variety of secure network programs, such as Android malware threat detection [6]. Most of the published solutions on Android device malware threat detection are described in the following section: Unver et al. [7] introduced an image visualization method for extracting locally and globally important malicious patterns from grayscale visuals in order to learn neural network-based classification algorithms to distinguish between legitimate and malicious Android apps. Chen et al. [8] and Hossain et al. [9] used an XGBoost model to classify Android malware images as either malicious or benign. Ding et al. [10] produced MixDroid to detect Android malware by combining multiple features and machine learning models using a bagging approach. Gu et al. [11] provide runnable adversarial cases for machine learning models based on malware visualization. Numerous works have been conducted on the detection of Android malware by CNNs. CNNs are set up to perform exceptionally well in image processing. Unpacked and packed malware can be automatically categorized using features learned from malware images. Researchers [10,12] and [13,14] used CNN-based training models to classify malicious apps using the bytecode of classes.dex file of Android application. When it comes to extracting images from DEX files, reference [15] used the same method, though they only used the data segment. Reference [16] created grayscale graphics by combining the data segment of DEX files with Android Manifest.xml entries. Such inputs are converted into a temporal convolutional network (TCN) in order to detect mobile malware. Lachtar et al. [17] used CNNs to identify malware on images obtained by space-filling curves from instinctive instructions in the app. Sun et al. [18] used CNNs to decompile an Android app, which was further transformed into pictures. Color images of Android application were used to train a ResNET for malware detection. In the layout used by Zhang et al. [19], capsule layers replace pooling layers in CNNs. In Chimera et al. [20], dense, convolutional, and textural neural networks were used to learn Android image patterns, for example, patterns of static permissions and authorizations and patterns of dynamic system calls. For cross-platform detection, Naeem et al. [21] used malware binary image information derived from combined CSGM characterization. Mercaldo et al. [22] proposed an Android malware classification system based on grayscale pictures taken from Android apps. Some studies had superior accuracy rates than the present introduced technique. Nevertheless, a straight contrast cannot be helpful because the samples utilized in that research are different. However, this may not function well with recent days malware vulnerabilities and can be unscalable when using feature engineering to choose hybrid features to attain excellent performance. When compared to SIFT or other feature extractors, the proposed technique provides the same benefits. N-gram technique worked well, but there is a significant computational cost associated with using it. Detecting zero-day attacks is critical in malware detection, and using CNN's structural features will save time and resources.

## 3. Proposed Approach

IoT device malware detection is divided into 2 phases: an explanation of how to turn Android bytecode into RGB and color images is given in Section 3.1 and the architecture of our proposed approach is explained in Section 3.2. Figure 1 shows the proposed explainable IoT device malware classification framework. First, a transfer learning mechanism is developed by combining global max pooling with dense activated layers. Second, fine-tuning is proposed by freezing a few blocks of the pretrained CNN model and retraining the remaining blocks. Third, Grad-CAM is used to produce cumulative heatmaps that aid the security analyst in interpreting the classifier selection. Finally, we learned large-scale data with GNB, K-NN, SVM, DT, and LR classification methods, as well as the ensemble classification method in conjunction with CNN models, to assess efficacy on IoT device malware classification.

### 3.1. Data Acquisition

#### 3.1.1. R2-D2 IoT Device Dataset [23]

It includes RGB pictures that were transformed from the DEX file collected by unpacking over two million Android malware and benign applications. Between January 2017 and August 2017, the original back-end detection system of Leopard Mobile Inc. acquired these apps. Trojans, Ad-Ware, Clickers, and SMS Spyware were among the types of malwares detected in the infected apps. In comparison to 8-bit grayscale images, the Android color image dataset allows for the storage of more crucial information about Android apps with 16777216 colors per image. Computer vision models can be trained quicker and more efficiently by reducing images to 299 × 299 pixels, which enables minibatch learning practicable. The images are between 10 and 50 KB in size.

**Figure 2:**
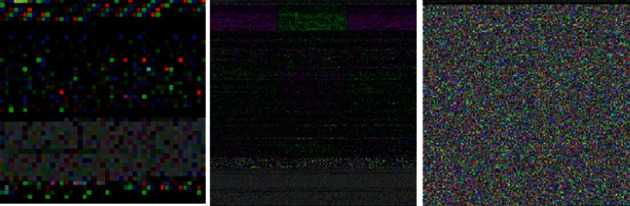
R2-D2 IoT device dataset.

*(1) RGB Image Representation*. The classes.dex file is obtained by first decompressing the Android APK. There is hexadecimal representation for both bytes and RGB colors in this file. RGB color coding is used to convert hexadecimal from the DEX files so that three-digit numbers are separated from one another in left-to-right sequence. A decimal code R, G, or B can be assigned to each of these integers in this series. For instance, 646778 was split into 64, 67, and 78, which were then transformed to R: 100, G: 103, and B: 120. RGB images are taken as the input of CNNs to identify malware on mobile platforms.  Figure 2 shows the chunks of malware images from the R2-D2 IoT device dataset.

#### 3.1.2. MalNet IoT Device Dataset [24]

MalNet has provided 8633 IoT device malware samples of 19 families. The sample distribution of each training malware dataset family is as follows: addisplay (1022), addisplay + + adware (59), adload (67), adsware (530), adware + + adware (501), adware + + grayware + + virus (167), adware + + virus (55), backdoor (121), banker + + trojan (221), adwareare (46), clicker (53), click (22), clicker + + trojan (573), clickfraud + + riskware (74), exploit (1116), fakeangry (42), fakeapp (85), fakeapp + + trojan (51), and fakeinst + + trojan (143), respectively.

**Figure 3:**
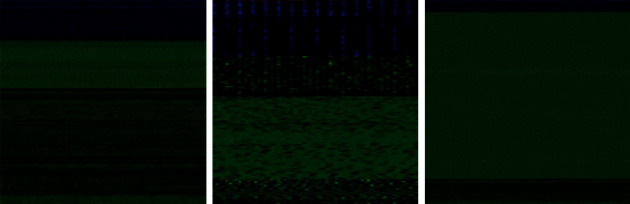
MalNet IoT device dataset.

*(1) Color Image Representation*. Semantic feature integration is a time-consuming process. Semantics can be extremely useful when it comes to analyzing bytecode. An ascii character, an opcode, or a piece of an address might all be represented by a single random byte. An additional layer of semantic information is added to the raw bytecode by using color to distinguish each byte according to its function. The encoding of semantic information into an image can be done in a variety of ways, but there is no established standard for doing so at this time. We are encoded the contextual features by allocating each byte to a specific RGB color channel based on its position in the DEX file structure: (i) header, (ii) signifiers and class interpretations, and (iii) data and by encoding the spatial meaning in binary form (Figure 3). The first step in creating a feature representation of Android applications and labels is to extract the DEX (bytecode) from each app. One dimensional array of eight-bit unsigned numbers is then generated from the DEX file. Black pixels and white pixels are represented by the values in a range of 0–255 in all of the entries. Each binary file goes through a three-step procedure that includes converting from a one-dimensional array to a two-dimensional feature extraction format, encoding data into RGB channels, and scaling images to the proper size.

### 3.2. Architecture of Transfer Learning-Based Pretrained CNN Model with Fine-Tuning

Figure 1 depicts the architecture design of proposed the transfer learning-based pretrained CNN model with fine-tuning. It is described in three parts below in detail.

#### 3.2.1. Transfer Learning-Based Pretrained CNN Model

When a neural network trained on a single dataset and task is transferred to a new issue with a different dataset and task, it is known as transfer learning. There is an exciting phenomenon shared by many deep neural networks trained on natural images. On the initial layers, these networks acquire general features that are not exclusive to a particular dataset or goal but apply to many datasets and tasks in general. Transfer learning might be a valuable technique to train an extensive target network without overfitting when the target dataset is considerably smaller. Our next step was to start using Inception-v3 [25], which has been pretrained to recognize objects in the ImageNet dataset. Malware image bottleneck features obtained by employing the convolutional layers of Inception-v3 served as input for training many different classification algorithms.

In the form of field, application, and marginal occurrence, the transfer learning of proposed IoT device malware classification framework is shown as follows: a field or domain “T” can be denoted as a set of two rows by {*y*, *P*(*Y*)}. Here, *y* is a feature space (i.e., *Y* = {*y*_1_, *y*_2_, *y*_3_,….,*y*_n_}€ *y*), and *P*(*Y*) represents marginal probability. Transfer learning cannot be applied to the two areas *y*_*s*_,*y*_*t*_ if they are too dissimilar. As a result, neither the feature space (*y*_s_ ≠ *y*_t_) nor the marginal occurrence distributions (*P*(*y*_s_) ≠ *P*(*y*_t_)) will overlap with one another.

(1) $$\begin{array}{c} T=\left\{\left\{y,P\left(Y\right)\right\}\right\}. \end{array}$$

An application or task “*D*” can also be shown as a set of two rows by {*X*, *f* (.)}. Here, *X* is a label and *f* (.) is a function for target prediction. Probabilistic expression of function *f*(.) is presented as follows:

(2) $$\begin{array}{c} D=\left\{\left\{X,f\left(.\right)\right\}\right\},\\ f\left(b_{i}\right)=\left\{P\left(\frac{a_{i}}{b_{i}}\right)\right\}, \end{array}$$

where *a*_i_ € *A*, *b*_i_ € *B* and *i* € {1,2,3, ...., *N*}.

Therefore, the task *D* can be expressed as follows:

(3) $$\begin{array}{c} D=\left\{\left\{X,P\left(\frac{A}{B}\right)\right\}\right\}. \end{array}$$

Consequently, if two tasks *D*_S_ and *D*_t_ are not the same, then they either have dissimilar label spaces or have dissimilar probability distributions.

**Figure 4:**
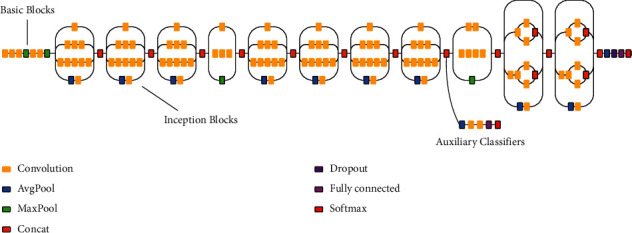
Inception-v3 architecture.

Figure 4 depicts the architecture of pretrained Inception-v3 model in schematic form. A convolution model based on Inception-v3 is used to sequentially combine the basic convolution block with the upgraded inception modules and the task-specific classifiers. Convolutional operations with 1 × 1 and 3 × 3 kernels are used to learn low-level feature mappings. Multiple scale feature representations are concatenated to feed into auxiliary classifiers with varied convolution kernels in the inception module, which is utilized to achieve better performance convergence. Image classification in conventional CNNs uses convolution, max-pooling, and two or more fully connected layers.

Nevertheless, most parameters are found in the fully connected layers, which might limit the model's generalizability and lead to overfitting. As a result, we added a global max-pooling (GMP) layer to reduce the prior layer's dimensions. Besides shrinking in size, GMP may also function as a regularizer by preventing some overfitting for the overall network structure. We added two fully connected layers with 100 and 50 lengths before our 19 or 2 states classifier. In our transfer learning model, these two fully connected layers are proceeded by a last fully connected layer with SoftMax function.

#### 3.2.2. Model Two Stage Training and Fine-Tuning

Models that have been previously trained on a large dataset, such as ImageNet, are called pretrained models. Transfer learning for image classification is based on the idea that if a model is trained on a big and diverse dataset, then it will serve as a general model for the visual world. Because the basic features of an image are extracted by the top layers of a pretrained model, fine-tuning the weights of upper layers is unnecessary. The weights of the lowest layers need to be fine-tuned in order to train them on a particular IoT device malware dataset. Hence, two-stage training was conducted for fine-tuning of proposed transfer learned model.

The first step trained the top layers of our transfer learned model, including the layers added on top of the Inception-v3 base model and the 19 states classifier. Before going on to the second stage, this step was used to correctly train the randomly initialized weights on these top layers to a sufficient level of performance and stability. In addition, the Inception-v3 base model layers were frozen because these weights had previously been well-trained on the ImageNet dataset. To begin the fine-tuning process, we used trained weights instead of a combination of pretrained and random weights in this first stage. The optimizer chosen for our first stage training was RMSprop:

(4) $$\begin{array}{c} \text{RMS}{\left[\Delta \theta \right]}_{t}⟵\sqrt{E{\left[\Delta \theta^{2}\right]}_{t}+\cup^{\prime }}. \end{array}$$

The second training phase begins when the top layer weights have been adequately trained. Some layers from our base model were unfrozen, and their pretrained weights were trainable throughout the fine-tuning stage. We found that fine-tuning the top two inception blocks (freezing layers up to the 249th layer) increased our model's overall accuracy. We kept the weights at the bottom layers of the Inception-v3 model frozen in both phases. These lower convolutional blocks extracted fundamental aspects of images such as shapes, edges, contours, and textures, which were also essential for malware classification challenges. SGD was chosen as the optimizer for our second stage of fine-tuning training; we discovered that because it is not an adaptive algorithm like adam or RMSprop, it allows for slower convergence. In other words, SGD is ideal for fine-tuning since it delivers minor updates to weights that have previously been trained. Some crucial properties of image categorization may be lost if weights are changed sufficiently by an adaptive learning system. The SGD optimization is expressed by the following equation:

(5) $$\begin{array}{c} \theta =\theta -\eta \nabla_{\theta }J\left(\theta ;x^{\left(i\right)};y^{\left(i\right)}\right). \end{array}$$

#### 3.2.3. Classification

*(1) SoftMax*. A single value is produced for each node in the output layer of the SoftMax activation function. It receives a vector *z* of K real numbers and transforms it into a probability distribution with K probabilities proportional to the exponentials of the input values. SoftMax function *σ* is expressed by the following formula:

(6) $$\begin{array}{c} \sigma {\left(z\right)}_{i}=\frac{e_{z_{i}}}{\sum_{j=1}^{k}e_{z_{i}}}, \end{array}$$

where *i* = 1………., *K* and *z*= (*z*_1_,,,,,,,,,*z*_k_) € *Ɍ*_*K*_.

*(2) Naive Bayes (NB)*. It is used to solve classification tasks. There are numerous circumstances, in which the Naive Bayes algorithm performs excellently since it is simple to grasp. Thefollowing equation states that the classifier is constructed using the Bayes theorem:

(7) $$\begin{array}{c} P\left(y|X\right)=\frac{P\left(X|y\right)P\left(y\right)}{P\left(X\right)}. \end{array}$$

In the equation, *y* is the class variable, while *X* is the characteristic or attribute. *X* is referred as *x*_1_, *x*_2_,…, *x*_n_. The Naive Bayes classifier assumes that attributes are unrelated as shown in the following equation:

(8) $$\begin{array}{c} P\left(y|X\right)=\frac{P\left(x_{1}|y\right)P\left(x_{2}|y\right)\ldots P\left(y\right)}{P\left(x_{1}\right)P\left(x_{2}\right)\ldots P\left(x_{n}\right)}. \end{array}$$

The following equation shows how a normal distribution is used to obtain the conditional probability in Gaussian Naive Bayes:

(9) $$\begin{array}{c} P\left(x_{1}|y\right)=\frac{\left(1/\sigma_{y}\sqrt{2\pi }\right)e^{-{\left(x_{i}-\mu_{y}\right)}^{2}}}{2\sigma_{y}^{2}}. \end{array}$$

*(3) Support Vector Machine (SVM)*. It uses supervised learning through the regression method. Classification is achieved by determining the hyperplane that most delineates the various groupings. It locates the hyperplane by increasing the space between the two points. The kernel trick technique transforms a nonseparable job into a separable solution by using the kernel function to turn a low-dimensional input vector into a higher-dimensional one. It is most advantageous when dealing with nonlinear discrete tasks. We used sigmoid as the kernel function:

(10) $$\begin{array}{c} \left[\frac{1}{n}\sum_{i=1}^{n}\max\left(0,1-y_{i}\left(w^{T}x_{i}-b\right)\right)\right]+w^{2}. \end{array}$$

*(4) Decision Tree (DT)*. It is a tree-structured data flow diagram, in which each leaf node represents the outcome, a branch represents a decision rule, and an interior node denotes a function or attribute. A decision tree has a root at the very top when making a choice. It tends to segment based on an attribute's value. Segmenting the tree iteratively is referred to as iterative segmentation. As a result, decision trees are easy to understand and learn. Tree-based decision-making evaluates division based on node purity and loss functions:

(11) $$\begin{array}{c} \text{Entropy}=-\sum_{i=1}^{K}p_{i}*\;\log_{2}p_{i}. \end{array}$$

The entropy value is a number that varies from 0 to 1.

*(5) Logistic Regression (LR)*. It assesses binary outcomes (*y* = 0 or 1) with high accuracy. Logistic regression is preferred over linear regression for predicting categorical outcomes (binomial/multinomial values of *y*). Hence, logistic regression is better at predicting continuous outputs. Equation (12) shows the mathematical representation of the logistic function:

(12) $$\begin{array}{c} fx=\frac{1}{1+e^{-x}}. \end{array}$$

*(6) Random Forest (RF)*. It [26] is an estimator that includes several distinct decision tree classifiers into its model to increase its forecasting supremacy and impact overfitting. Decision trees are typically taught using the “bagging” approach, which results in a “forest” of trees. The bagging technique is based on the belief that combining many learning models will yield superior results.

*(7) K-Nearest Neighbour (K-NN)*. It is a simple method based on the local minimum of the target function, which is used to learn an unknown process with desired precision and accuracy. In addition, the approach determines a parameter's range and distance from an unknown input. The algorithm uses the “information gain” concept to determine which data points are most likely to forecast an unknown value.

**Figure 5:**
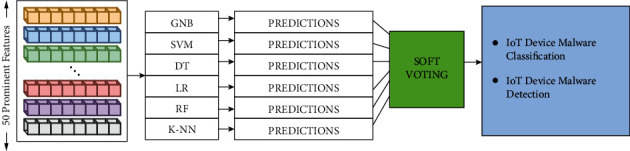
Proposed ensemble learning.

*(8) Ensemble Classifier*. It [27] is a robust model constructed by carefully combining base classification methods. The ensemble model's mix of methodologies can solve classification and regression difficulties that any individual model cannot handle. It is possible to outperform single models via ensemble learning. The proposed study includes a soft voting ensemble approach. GNB, SVM, DT, LR, KNN, and RF were developed using training data for the basic models. We then utilized test data to evaluate the performance of our models, with each model predicting a different outcome. The ensemble learning approach combines the estimations provided by various techniques to come at the final classification results.  Figure 5 depicts the proposed ensemble learning.

## 4. Results and Discussion

Besides our transfer learning-based pretrained fine-tuned CNN model, three other state-of-the art CNN pretrained models for IoT device virus classification will be used in this study's experimentation. These three pretrained CNN models were ResNET50, MobileNETV2, and DenseNET201. Pretrained models in the Keras Applications were run for 50 epochs on the malware dataset with a batch size of 32. These models' accuracies in detecting malware on IoT devices and losses are listed in Table 1 for datasets 1 and 2 (as depicted). Experiments were conducted using an NVIDIA GeForce GTX 2060 6 GB GPU and 16 GB of main memory.

### 4.1. Performance Metrics

A confusion matrix was used to evaluate the various models that were considered and compared. Classifier predictions are summarized in an error matrix, namely, a confusion matrix. False-positive (FP), false-negative (FN), and true-positive (TP) values are all provided by the confusion matrix. A malicious program (TP) and a nonmalicious program (TN) are two distinct concepts in this work. False malware samples are counted as FP values, and the number of false malware samples is counted as FN values. We calculated the following performance metrics with these values. Accuracy refers to the ability of a classifier to differentiate all positive instances as correct and all negative instances as wrong:

(13) $$\begin{array}{c} \text{accuracy}=\frac{\text{TP}+\text{TN}}{\text{TP}+\text{TN}+\text{FP}+\text{FN}}. \end{array}$$

Precision refers to a classifier's capacity to avoid classifying a negative instance as positive:

(14) $$\begin{array}{c} \text{precision}=\frac{\text{TP}}{\text{TP}+\text{FP}}. \end{array}$$

Recall measures the classifier's capacity to identify all positive instances correctly:

(15) $$\begin{array}{c} \text{recall}=\frac{\text{TP}}{\text{TP}+\text{FN}}. \end{array}$$

Precision and recall's weighted average is F1-score:

(16) $$\begin{array}{c} F-\text{measure}=2*\;\frac{\text{Precision}*\text{Recall}}{\text{Precision}+\text{Recall}}. \end{array}$$

### 4.2. Performance Analysis

To validate the proficiency of the proposed transfer learning-based pretrained fine-tuned CNN model, we conducted experiments to (1) verify the impact of fine-tuning on classification performance, (2) validate the effectiveness of deep features with the performance of machine learning, and (3) performance compared with previous works.

#### 4.2.1. Impact of Fine-Tuning on Classification Performance

Four different CNN pretrained models for the IoT device malware identification are listed in Table 2 with their train accuracy, test accuracy, train loss, and loss performance metrics. Table 1 summarizes the model's accuracy results on datasets 1 and 2. SoftMax classification activation function was used to obtain probabilistic predictions on datasets 1 and 2. Table 2 highlights the classification accuracies of the transfer learning-based pretrained fine-tuned Inception-v3 model and baseline CNNs. The proposed technique outperformed all other CNN models regarding classification accuracy. The accuracy of the proposed approach was 0.969 on dataset 1 and 0.786 on dataset 2, respectively, which was greater than that of the baseline methods (transfer learned DenseNET201, MobileNET, and ResNET50). Hence, it can be seen that the addition of fine-tuning with transfer learning significantly improves the overall testing accuracy of the pretrained Inception-v3 model. The epoch curve can examine the dynamic behavior of accuracy and loss values. This enables us to quickly identify the various patterns of categorization values and overfitting. The accuracy and loss epoch curves for training and testing data are analyzed to demonstrate the feasibility of the proposed approach.

**Figure 6:**
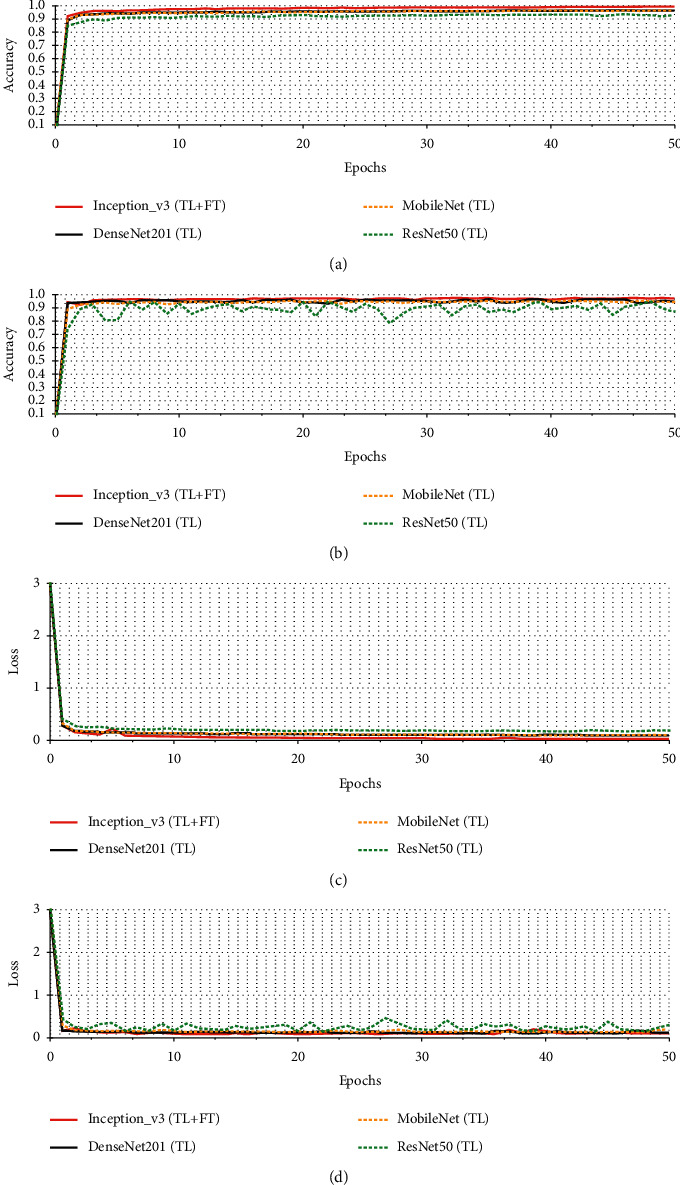
Training and test curves of four different CNN pretrained models on dataset 1. (a) Training accuracy. (b) Testing accuracy. (c) Training loss. (d) Testing loss.

**Figure 7:**
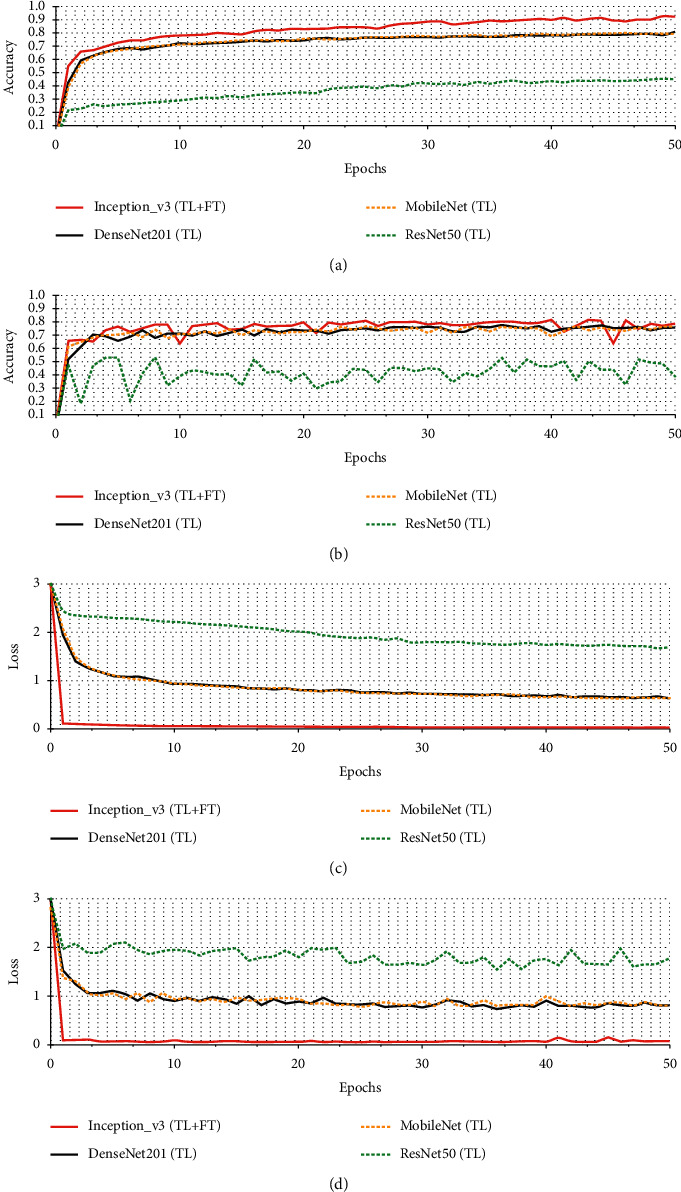
Training and test curves of four different CNN pretrained models on dataset 2. (a) Training accuracy. (b) Testing accuracy. (c) Training loss. (d) Testing loss.

Figure 6 shows the accuracy and loss curves of the four CNN models on dataset 1. The *x* axis shows the epoch values, while the *y* axis shows the corresponding accuracy values. The experimental data from the first 50 epochs are utilized to clear the display of any information. Pretrained Inception-v3 (transfer learning with fine-tuning) accuracy and loss are depicted by the solid red lines on training and test dataset 1. Figures 6(a) and 6(b) show that the proposed model's training and testing accuracies converge fast as the number of epochs increases. The proposed pretrained Inception-v3 model's training and testing accuracies converge faster than the other three pretrained CNN models. All three pretrained CNN models have testing accuracies of roughly 0.947, 0.942, and 0.868 when the training accuracy is steady. However, pretrained Inception-v3' (transfer learning with fine-tuning) testing accuracy ranges from 0.955 to 0.969%. Figures 6(c) and 6(d) show the training and test loss epoch curves for the pretrained Inception-v3 (transfer learning with fine-tuning) and three different pretrained CNN models. In Figure 6(c), ResNET50 has the worst training loss, with values ranging from 3 to 0.18. The pretrained Inception-v3 (transfer learning with fine-tuning) has the lowest training loss, indicating that it performs the best categorization. When the training loss curve reaches 3, it declines slowly to 0.3. After that, it acted reasonably predictable manner up to the fourth epoch. In the fifth epoch, the loss rises to 0.28. Compared to other state-of-the-art techniques, it works within a range of 3 to 0, which is the best-estimated train loss. Similarly, in Figure 6(d), ResNET50 has the more testing loss, with values ranging from 3 to 0.3. The pretrained Inception-v3 (transfer learning with fine-tuning) has the lowest testing loss, indicating that it performs well for the classification task. When the testing loss curve reaches 3, it declines slowly to 0.3. After that, it acted reasonably predictable up to the fourth epoch. In the fifth epoch, the loss rises to 0.29. Compared to three other state-of-the-art pretrained CNN models, it acts within a range of 3 to 0.1, which is the best-estimated test loss.  Figure 7 shows the accuracy and loss curves of the four CNN models on dataset 2. Figures 7(a) and 7(b) show that the proposed model's training and testing accuracies overlap fast as the number of epochs increases. In Figure 7(a), ResNET50 has less training accuracy, with values ranging from 0.1 to 0.44. The pretrained Inception-v3 (transfer learning with fine-tuning) has the highest training accuracy, indicating that it performs well for the classification task. When the training accuracy curve reaches 0.1, it increases slowly to 0.922. Compared to other state-of-the-art techniques, it acts within a range of 0.1 to 0.9, which is the best training accuracy. In Figure 7(b), ResNET50 has less testing accuracy, with values ranging from 0.1 to 0.38. The pretrained Inception-v3 (transfer learning with fine-tuning) has the highest testing accuracy, indicating that it performs better for classification. When the testing accuracy curve reaches 0.1, it increases slowly to 0.786. Even though the proposed model does not correctly overlap train and test data as the number of epochs increases, it nonetheless delivers less data overfitting than three different CNN pretrained models. Figures 7(c) and 7(d) show the training and test loss epoch curves for the pretrained Inception-v3 (transfer learning with fine-tuning) and three different pretrained CNN models. In Figure 7(c), ResNET50 has the worst training loss, with values ranging from 3 to 1.686. The pretrained Inception-v3 (transfer learning with fine-tuning) has the lowest training loss, indicating the best categorization results. When the training loss curve reaches 3, it declines slowly to 0.1. Compared to other state-of-the-art techniques, it acts within a range of 3 to 0, which is the best-estimated train loss. Similarly, in Figure 6(d), ResNET50 has the more testing loss, with values ranging from 3 to 1.779. The pretrained Inception-v3 (transfer learning with fine-tuning) has the lowest testing loss, indicating that it performs well for the classification task. When the testing loss curve reaches 3, it declines slowly to 0.1. Compared to three different CNN pretrained models, it acts within a range of 3 to 0, which is the best-estimated test loss.

As a result, the high training and testing accuracy curves of the proposed model show that the data were well-trained throughout learning. Fine-tuning and transfer learning considerably minimize the risk of overfitting with an epoch increase.

#### 4.2.2. Effectiveness of Deep Features with the Performance of Machine Learning Classifier

When it comes to classification, feature engineering is crucial. Image processing methods used for feature extraction are inefficient and prone to error. We employed a transfer learning-based pretrained fine-tuned Inception-v3 model to extract 50 prominent features for IoT device malware classification since the features for IoT malware from color images are complicated. The dataset was delivered to the trained CNN model when the learning phase was complete. The 50 most prominent features of each malware image were retrieved from the second last dense layer, which had 50 neurons. Large-scale learning classifiers including GNB, SVM, DT, LR, K-NN, and an ensemble classifier were used to replace the SoftMax layer in the proposed approach. Accuracy, F1-score, precision, and recall were used as standard performance measurements. For dataset 1, we found that the proposed method with random forest performed well with an accuracy of 0.985, F1-score of 0.99, precision of 0.99, and recall of 0.99. Similarly, for dataset 2, we found that the proposed method with random forest performed well with an accuracy of 0.91, F1-score of 0.91, precision of 0.91, and recall of 0.91). The detailed results on both datasets are presented in Table 3.

The proposed model's overall and class-wise performance in both IoT device malware datasets is listed in Table 4. A high proportion of recall reflects successfully classifying instances in both datasets. The results indicate that the true positive ratio for both dataset 1 classes was high. Consequently, the proposed model had the highest rate of detection (98.5%). Compared to other classes in dataset 2, the recall percentage for clicker++trojan and exploit was lower (87%) as there was more misunderstanding in samples from the clicker, clickfraud++riskware, fakeangry, and fakeapps classes. A maximum classification rate of 91% was achieved using dataset 2.

**Figure 8:**
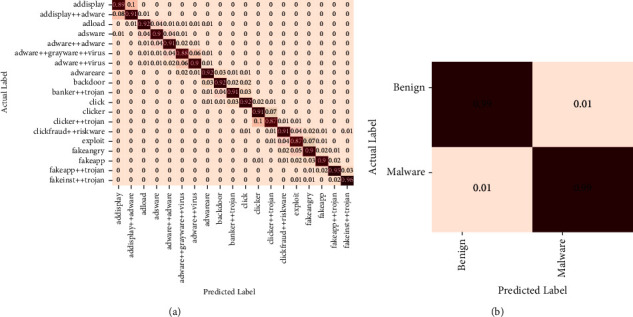
Confusion matrices of both IoT device malware datasets for the proposed model.

Table 4 lists that the proposed model could assist security analysts in detecting IoT device malware with a lower error rate.  Figure 8 shows the proposed model's confusion matrices for both IoT device malware datasets. Accordingly, each class's actual and predicted labels are shown vertically and horizontally. In two normalized confusion matrices, the prediction accuracies of instances were presented. In confused matrices, normalized detection and classification rates were shown. The first dataset's confusion matrices revealed a malware detection rate of 0.99, similar to the benign detection rate of 0.99. It revealed that the sample distribution in the two classes was not biased. The second dataset's confusion matrices revealed that the clicker + + trojan and exploit families had the lowest classification rates of 0.87 compared to other malware families. The family fakeinst + + trojan received the highest classification rate of 0.96.

#### 4.2.3. Performance Comparison with Previously Published Works

We compared the suggested malware classification technique with previous published studies in terms of detection accuracy. Yen et al. [18] used CNNs to decompile a mobile app into code, subsequently visualized using the TF-IDF method. Color graphics of Android platform modules were utilized for training ResNET networks to identify attacks. As opposed to pooling layers, Farhan et al.'s DCNN approach uses capsule layers [28]. Chimera et al. [20] utilized images that had been converted to DEX files, as well as Android purpose and intent and system call trends. Dense, convolutional, and textural neural networks were employed to discover patterns from various sorts of data. Naeem et al. [21,29] utilized malware binary image data from the SIFT-GIST (CSGM) description to locate malware that can execute on different systems. Mercaldo et al. [22] created a way for classifying malware based on grayscale images captured by mobile apps.  Table 5 summarizes the models, characteristics, and datasets employed in the aforementioned publications. The malware detection accuracy is also mentioned in the table based on these works on their respective IoT malware datasets. Some research outperformed the suggested method in terms of accuracy. A straight comparison may not be effective due to the diversity of datasets used in these studies. It is feasible to obtain good outcomes with feature engineering, but it may not be scalable and may not work well with recent days malware vulnerabilities. When compared to SIFT or other feature extractors, the proposed method has the same benefits. As a result, the proposed transfer learning-based pretrained fine-tuned Inception-v3 model produces more accurate results than typical handcrafted features.

## 5. Performance Distribution

### 5.1. Feature Interpretation

**Figure 9:**
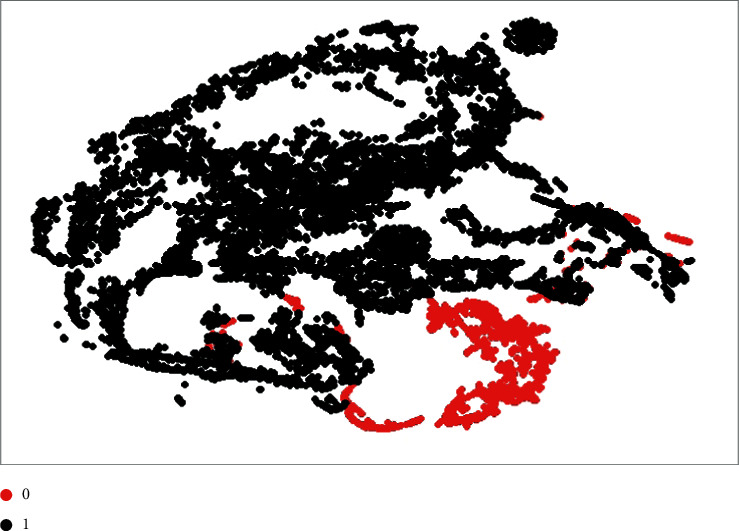
Two-dimensional pattern representation of the suggested model on the IoT device malware dataset (“0”: benign and “1”: malware).

To ensure the superiority of the learned patterns, we used the t-distributed stochastic neighbor embedding (t-SNE) [31] approach to display the image representations contained in the features of the IoT device malware dataset. High-dimensional data points are fed into the t-SNE algorithm, which depicts the points exactly in the low dimension domain. It aims to preserve both the local and global structure of the data, such as data clusters. Furthermore, it can attain such high performance while having limited processing resources. We used t-SNE to generate a two-dimensional representation of the characteristics retrieved from the second-to-last dense layer. t-SNE tuning parameters are perplexity = 100.0 and a number of iterations = 700. Feature representation in two dimensions for the IoT device malware dataset is shown in Figure 9. The figure shows that the suggested model does a remarkable job of distinguishing between “benign (0)” and “malware (1)” data instances.

### 5.2. Explainable Model

Two important experiments, such as explainable AI and t-SNE visualization, are carried out to validate the proposed approach. To generate more accurate findings, we used the gradient-weighted class activation mapping (Grad-CAM) approach to locate the model's most valuable regions of the image. Consequently, the security analyst will see which portions of the IoT malware image are predictive of a particular forecast. We also designed a cumulative heatmap to aid security analysts in understanding the classifier's results. The cumulative heatmap [9], a graphical depiction of model performance, can give security analysts quick insight into the model's behavior. To make greater use of Grad-CAM, security analysts may employ automated heat map analysis to troubleshoot models without prior knowledge of the problem or patterns. The following stages outline the explainability of the proposed transfer learning-based pretrained fine-tuned Inception-v3 model.

#### 5.2.1. Heatmap

When it comes to explaining deep neural networks, the heatmap generated by integrated gradients is one of the most used methods. It tells you which pixels are most crucial for making predictions. A security analyst may inspect the machine's pixels to discover the exact malware attack and its patterns. The model classifies a subset of the test dataset samples based on these characteristics. As part of the inference process, Grad-CAM analyses the pictures to determine which areas are most suited for categorization. Each instance in this subgroup is shown using a heatmap.

#### 5.2.2. Cumulative Heatmap

The average value for each pixel is used to create cumulative heatmaps. We construct a heatmap that depicts “unique features” of that exact family. As a result of this, a security analyst will be able to investigate a large number of pixels for a certain malware attack more quickly and with less effort.

**Figure 10:**
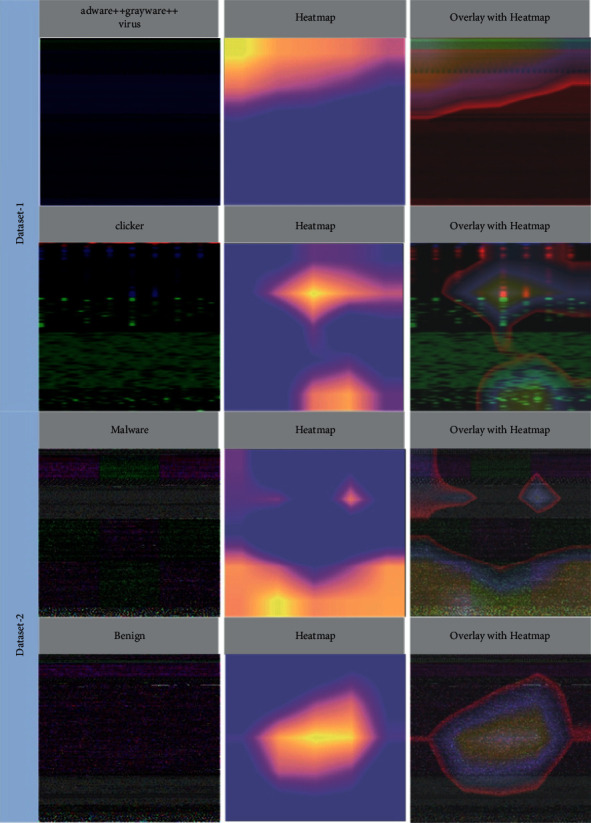
First column: original image; second column: heat map; third column: overlay with heatmap.

Figure 10 shows the original images from two separate IoT device malware datasets, the heatmap generated by the Grad-CAM, and the significance of the regions on the original image by overlaying the heatmap. Cumulative heatmaps are created by overlaying all heatmaps from the same family and applying pixel-by-pixel averages to create a new image. The final outcome is only an image containing information on the IoT device malware family's inferences.

## 6. Conclusions

A transfer learning-based pretrained fine-tuned Inception-v3 model is proposed in this study to identify IoT device malware. It can withstand both unpacked and packed malware, regardless of the platform on which it is run. First, it employs the IoT device malware image as input to the pretrained Inception-v3 network to extract critical characteristics. Second, the collected features are transfer learned via GMP and dense layers and inserted into a classification method. Third, the dual training of projected model is proposed in order to fine-tune weights. Finally, the SoftMax classifier distinguished between malicious and benign images with a precision of 96.9% on dataset 1. We evaluated the model with three different CNN-based pretrained models. SVM, RF, GNB, LR, KNN, and DT classifiers and ensemble classifiers with CNN models were used for large-scale learning. The proposed approach obtained maximum classification and detection accuracies of 98.5% and 91%, respectively, with RF on both available datasets. According to the findings, the proposed approach outperformed every indicator compared to the alternatives. Besides this, fine-tuning and transfer learning considerably minimize the risk of overfitting in the proposed method.

In the future, we intend to use principal component analysis to improve malware classification on IoT devices. Besides this, we intend to examine the suggested model on a number of large-scale public datasets of Android malware, such as Drebin and AndroZoo. It is possible to obtain numerous folds of training and testing data using cross-validation via deep neural networks. It can alleviate problems such as over- or underfitting.

## Figures and Tables

**Table 1:** Comparison of detection accuracy between four different CNN pretrained models.

Pretrained model	Total parameters	Total depth	Accuracy (dataset 1)	Accuracy (dataset 2)
DenseNET201	18,520,103	707	0.947	0.757
MobileNET	3,337,383	87	0.942	0.764
ResNET50	23,798,631	175	0.868	0.387
Inception-v3	22,012,734	311	0.969	0.786

**Table 2:** Comparison of accuracy and loss between four different CNN pretrained models.

Dataset 1				
Pretrained model	Train accuracy	Train loss	Test accuracy	Test loss
DenseNET201 (TL)	0.963	0.093	0.947	0.127
MobileNET (TL)	0.964	0.092	0.942	0.195
ResNET50 (TL)	0.929	0.18	0.868	0.303
Inception-v3 (TL + FT)	0.992	0.023	0.969	0.107

Dataset 2				
DenseNET201 (TL)	0.803	0.629	0.757	0.804
MobileNET (TL)	0.792	0.637	0.764	0.805
ResNET50 (TL)	0.449	1.686	0.387	1.779
Inception-v3 (TL + FT)	0.922	0.021	0.786	0.073

TL, transfer learning; FT, fine-tuning.

**Table 3:** Performance of deep features with different machine learning classifiers.

Dataset 1				
Classifier	Precision	Recall	F1-score	Accuracy
Decision tree	0.98	0.98	0.98	0.982
Logistic regression	0.9	0.9	0.9	0.896
Random forest	**0.99**	**0.99**	**0.99**	**0.985**
K-nearest neighbor	0.98	0.98	0.98	0.984
Ensemble learning	0.98	0.98	0.98	0.984

Dataset 2				
Decision tree	0.88	0.88	0.88	0.875
Logistic regression	0.25	0.27	0.25	0.273
Random forest	**0.91**	**0.91**	**0.91**	**0.91**
K-nearest neighbor	0.88	0.88	0.88	0.881
Ensemble learning	0.9	0.9	0.9	0.902

For dataset 1, we found that the proposed method with random forest performed well with an accuracy of 0.985, F1-score of 0.99, precision of 0.99, and recall of 0.99. Similarly, for dataset 2, we found that the proposed method with random forest performed well with an accuracy of 0.91, F1-score of 0.91, precision of 0.91, and recall of 0.91).

**Table 4:** Classwise performance.

Dataset 2			
Families	Precision	Recall	F1-score
Addisplay	0.91	0.89	0.9
Addisplay + + adware	0.89	0.91	0.9
Adload	0.92	0.92	0.92
Adsware	0.9	0.9	0.9
Adware + + adware	0.9	0.91	0.91
Adware + + grayware + + virus	0.88	0.88	0.88
Adware + + virus	0.91	0.9	0.9
Adwareare	0.92	0.92	0.92
Backdoor	0.92	0.92	0.92
Banker + + trojan	0.94	0.91	0.92
Click	0.92	0.92	0.92
Clicker	0.88	0.91	0.9
Clicker + + trojan	0.89	0.87	0.88
Clickfraud + + riskware	0.91	0.91	0.91
Exploit	0.86	0.87	0.86
Fakeangry	0.88	0.9	0.89
Fakeapp	0.94	0.9	0.92
Fakeapp + + trojan	0.96	0.95	0.95
Fakeinst + + trojan	0.96	0.96	0.96
Average	0.91	0.91	0.91

Dataset 1			
Benign	0.99	0.99	0.99
Malware	0.99	0.99	0.99
Average	0.99	0.99	0.99

**Table 5:** Comparison of the proposed model with previously published works.

Published works (year)	Detection model	Feature type	Data source	Accuracy (%)
Yen et al. [18]	Convolutional neural network	Code retrieved from APKs with image-based characteristics	—	92
Ullah et al. [28]	Deep convolutional neural network	Code retrieved from APKs with image-based characteristics	R2-D2 IoT device dataset	97.46
de Oliveira et al. [20]	Combined CNN + DNN + TN neural network model	Characteristics extracted from static and dynamic analyses	Omnidroid	90.90
Hamad et al. [21]	K-nearest neighbor	Code retrieved from APKs with image-based characteristics	R2-D2 IoT device dataset	97.29
Hamad et al. [29]	Deep convolutional neural network	Code retrieved from APKs with image-based characteristics	R2-D2 IoT device dataset and malimg	98
Mercaldo et al. [22]	Deep neural network	Code retrieved from binaries with image-based characteristics	Google play store and AMD	91.8
Yadav et al. [30]	Pretrained efficient net convolutional neural network	Code retrieved from binaries with image-based characteristics	R2-D2 IoT device dataset	95.7
Proposed approach	Pretrained Inception-v3	Code retrieved from APKs with image-based characteristics	R2-D2 IoT device dataset	98.5

## Data Availability

The data used to support the findings of this study are available from the first author upon request.
